# Anhydrous Phase B: Transmission Electron Microscope Characterization and Elastic Properties

**DOI:** 10.1029/2019GC008429

**Published:** 2019-08-14

**Authors:** A. Addad, P. Carrez, P. Cordier, D. Jacob, S.‐I. Karato, A. Mohiuddin, A. Mussi, B. C. Nzogang, P. Roussel, A. Tommasi

**Affiliations:** ^1^ Université de Lille, CNRS, INRA, ENSCL, UMR 8207 ‐ UMET ‐ Unité Matériaux et Transformations Lille France; ^2^ Department of Geology and Geophysics Yale University New Haven CT USA; ^3^ Université de Lille, CNRS, Centrale Lille, ENSCL, Université d'Artois, UMR 8181 ‐ UCCS ‐ Unité de Catalyse et de Chimie du Solide Lille France; ^4^ Université de Montpellier, CNRS, Geosciences Montpellier Montpellier France

**Keywords:** transmission electron microscopy, anhydrous phase B

## Abstract

- Anhydrous phase B and stishovite formed directly from olivine in experiments at 14 GPa and 1400 °C
- The structure of anhydrous phase B is determined ab initio from precession electron diffraction tomography in transmission electron microscopy
- Elastic and seismic properties of anhydrous phase B are calculated

## Abstract (fr)

We have performed an extensive characterization by transmission electron microscopy (including precession electron diffraction tomography and ab initio electron diffraction refinement as well as electron energy loss spectroscopy) of anhydrous phase B (Anh‐B) formed directly from olivine at 14 GPa, 1400 °C. We show that Anh‐B, which can be considered as a superstructure of olivine, exhibits strong topotactic relationships with it. This lowers the interfacial energy between the two phases and the energy barrier for nucleation of Anh‐B, which can form as a metastable phase. We have calculated the elastic and seismic properties of Anh‐B. From the elastic point of view, Anh‐B appears to be more isotropic than olivine. Anh‐B displays only a moderate seismic anisotropy quite similar to the one of wadsleyite.

## Plain Language Summary

Anhydrous phase B (Anh‐B) is a dense magnesium silicate with composition (Mg, Fe)_14_Si_5_O_24_, which is expected to form in Mg‐rich or Si‐depleted regions of the mantle. We show that due to strong crystallographic similarities with the crystal structure of olivine, it can form directly from it as a metastable phase. We show that Anh‐B exhibits a moderate seismic anisotropy, which makes its detection difficult in the mantle.

## 1 Introduction

The sequence of phase transformations of the major rock‐forming minerals with increasing temperature and pressure in the Earth's mantle is relatively well understood. The most abundant mineral in the upper mantle, olivine, transforms to wadsleyite at 410 km (approximately 14 GPa; Katsura et al., 2004), which then transforms to ringwoodite at 520 km (≈18 GPa near 1600 K; Katsura & Ito, 1989; Akaogi et al., 1989). The second most abundant mineral, orthopyroxene, undergoes gradual transformation (completed in the pressure interval 14‐16 GPa) to majorite garnet through the reaction with pyrope garnet (e.g., Irifune, 1987; Ringwood, 1991). Then ringwoodite and majorite react to form a mixture of bridgmanite and ferropericlase (at approximately 23‐24 GPa; Hirose, 2002). Through these phase transformations, the minerals change to denser crystal structures. These changes are sometimes associated with a change in the coordination of oxygen ions surrounding a cation, in particular silicon (Finger & Hazen, 1991).

In these two‐phase transformation sequences, the (Mg+Fe)/Si ratio is between 1 and 2 (1 for orthopyroxene and 2 for olivine). Although these two sequences explain a majority of seismological observations, the role of some minor phases is often invoked to explain some geological observations. An important case is the anhydrous phase B (Anh‐B), which has an unusually high (Mg+Fe)/Si ratio (2.8; Finger et al., 1991). For example, Ganguly and Frost (2006) investigated the stability of Anh‐B and suggested that it be an explanation of the so‐called X discontinuity that has been reported at 275‐ to 345‐km depth in several subcontinental and subduction zone environments. Yuan et al. (2018) extended such a study to higher pressures and proposed that Anh‐B could offer an alternative interpretation of the origin of the paragenesis of olivine and periclase found in some natural diamonds.

However, in both studies, the stability of Anh‐B phase relative to olivine (or wadsleyite or ringwoodite) + MgO was studied in a system with a very high Mg/Si ratio (=2.8). In the real Earth, (Mg+Fe)/Si is lower (=1‐2) and a more likely reaction is the formation of Anh‐B and stishovite from olivine (or wadsleyite or ringwoodite). However, the details of this reaction remain unclear. Ganguly and Frost (2006) calculated the thermodynamic parameters of Anh‐B and predicted that Anh‐B + stishovite might be present in a cold slab. However, this prediction has not been tested experimentally.

In this study, we report an extensive characterization by transmission electron microscopy (TEM) of lamellae of Anh‐B formed in a sample of polycrystalline olivine annealed at 14 GPa, 1400 °C. Structure refinement is performed from processing three‐dimensional precession electron diffraction data. The structure model is complemented by chemical analysis by energy‐dispersive X‐ray spectroscopy (EDS), and silicon coordination is characterized by electron energy loss spectroscopy (EELS) analyses. From this structure model, first‐principles atomic scale calculations are carried out to investigate the stability of this phase and to determine its elastic properties, and then its seismic properties.

## 2 Experiments and Microstructural Characterization

### 2.1 High‐Pressure Synthesis

Large single crystals of San Carlos olivine with no visible inclusions were handpicked and crushed to make a fine‐grained powder by cold pressing in a hydraulic press. The powder was then sorted to obtain ~5‐ to 10‐μm grains by suspension method. The powder was then cold pressed in a hydraulic press and then vacuum sintered in a furnace oven at 1200 °C for 12 hr to obtain a dense polycrystalline aggregate of San Carlos olivine. The dense olivine aggregate was then core drilled to obtain a cylindrical sample of 1.8‐mm length and 1.8‐mm diameter.

High‐pressure and high‐temperature experiments were carried out in a KIWI 1000‐ton Kawai‐type multianvil apparatus installed at Yale University. A 10‐mm edge length Cr_2_O_3_‐doped MgO octahedron with 5‐mm truncation edge length of WC anvils was used for pressure generation. A 40‐μm‐thick rhenium foil was used as a heater, which was inserted in a ZrO_2_ thermal insulation sleeve for heating. A W_5_Re‐W_26_Re thermocouple was used to monitor the temperature. All the ceramic parts were fired at 1000 °C for 12 hr to remove adsorbed water prior to high‐pressure experiments.

The pressure was calibrated as function of hydraulic oil pressure using the experimental results on the olivine‐wadsleyite transformation (P = 14 GPa at T = 1400 °C) by Katsura et al. (2004). The temperature distribution in the cell assembly was mapped using the enstatite‐diopside thermometry at 15 GPa (Gasparik, 1996). Temperature within the sample did not vary by more than ~30 K. In the experiment reported here, pressure was increased to 14 GPa over a period of 5 hr. After reaching the desired pressure, temperature was increased rapidly to 1400 °C (>100 °C per min). The sample was then annealed at 1400 °C for 30 min.

**Figure 1:**
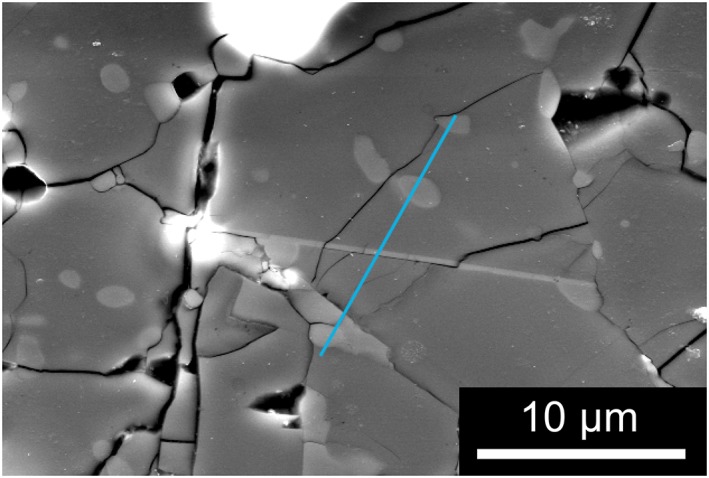
Scanning electron microscopy examination of the run product of experiment K1487 annealed at 14 GPa, 1400 °C for 30 min. Transformation products appear in light gray as either irregular domains or planar lamellae, which are of interest in this study. The blue line shows the location of focus ion beam section, which crosscuts a well‐developed lamella.

Scanning electron microscopy (SEM) examination of the recovered sample showed that olivine was partially transformed into denser (appearing brighter in backscattered mode) phases with two distinct morphologies. Intragranular or intergranular domains with irregular but often rounded shapes and some thin planar lamellae (Figure 1).

### 2.2 Microstructural, Structural, and Chemical Investigation by TEM

#### 2.2.1 Sample Preparation

Electron‐transparent foils for TEM containing the lamellae were extracted using the focus ion beam (FIB) technique with a FIB Dual Beam FEI Strata DB 235 at IEMN‐Lille, France. Figure 1 shows the location in the sample of one of the FIB sections. The thin section was lifted off below the line, perpendicular to the surface.

#### 2.2.2 Microstructural Investigation

**Figure 2:**
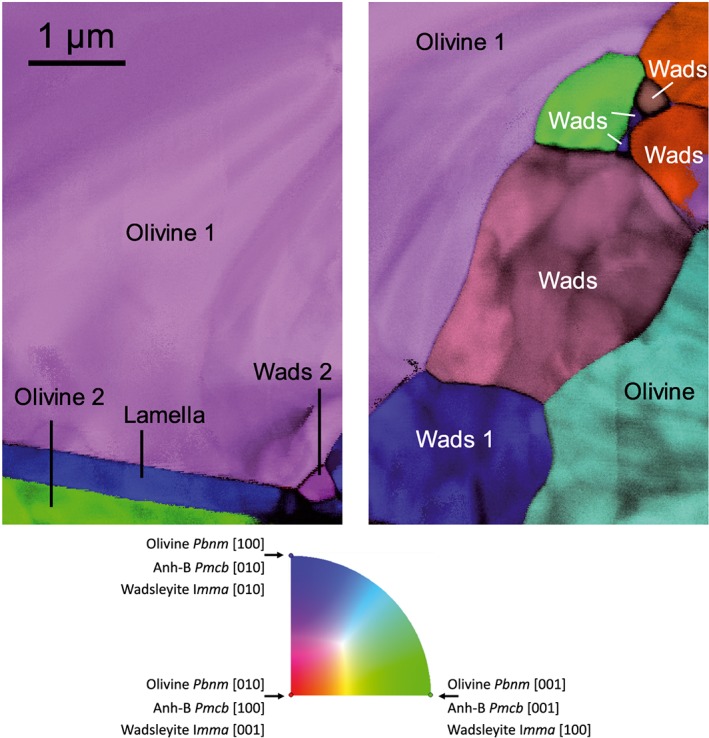
Two adjacent (with a small gap in between) orientation maps from the focus ion beam foil extracted from the location indicated on Figure 1. Spatial resolution: 10 nm. 550 × 350 data points. Precession angle 0.5°. The inverse pole figures represented here correspond to the vertical direction. They are superimposed with the reliability maps. Additional inverse pole figures (corresponding to the vertical and normal‐to‐plane directions), as well as the phase map, are provided in the supporting information. The color code is provided for the crystal orientations of the three phases.

TEM observations were carried out using a FEI® Tecnai G2‐20 twin microscope operating at 200 kV and a Philips CM30 microscope operating at 300 kV, both equipped with a LaB_6_ filament and using a double tilt sample‐holder. Automated crystal orientation mapping in TEM (ACOM‐TEM) was operated in the TEM with the ASTAR^TM^ tool from NanoMEGAS. As on SEM images, one can observe within and at the limits of olivine crystals irregularly shaped grains, which are indexed with ACOM‐TEM as wadsleyite, and a planar lamella located at the boundary between two olivine grains (Figure 2). With ACOM‐TEM, the lamella can be indexed neither as olivine nor as wadsleyite.

**Figure 3:**
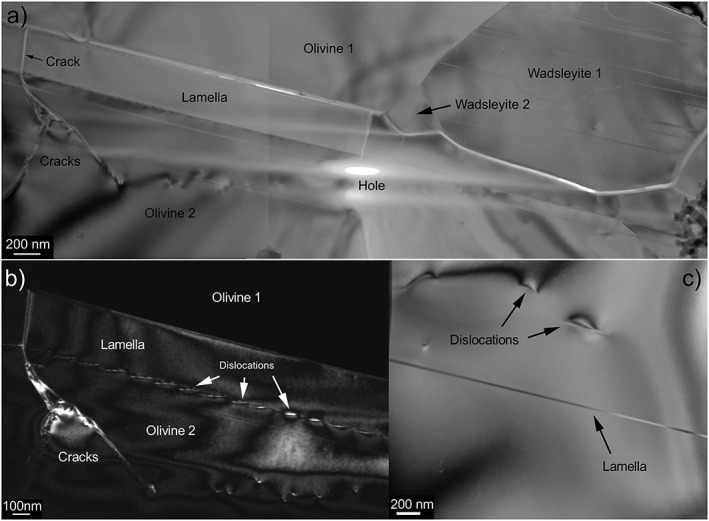
Conventional (diffraction contrast) transmission electron microscopy image of the area containing the lamella in the focus ion beam section, which location of extraction is shown in Figure 1. (a) Bright field image showing the lamella between two olivine grains and next to two wadsleyite grains. This area is mapped at the bottom of Figure 2. The small hole results from the ion thinning process. Several cracks are visible which probably result from decompression and/or specimen thinning. (b) Weak‐beam dark‐field image (**g** = 004 for olivine and **g** = 400 for anhydrous phase B) showing that the lamella and the olivine 2 grain are in topotactic relationship and exhibit a network of misfit dislocations at the interface. (c) Bright‐field image from the “olivine 2” grain showing one of the thin lamellae observed within this grain.

In diffraction contrast (Figure 3), wadsleyite displays characteristic planar defects in (010). The lamella is free of defects. It exhibits a crystallographic relationship with one of the olivine grains illustrated in Figure 3b where both the lamella and olivine grain 2 show similar contrast in weak‐beam dark‐field. A network of misfit dislocations is observed at the interface between the two phases (Figure 3b). This lamella located at the boundary between two olivine grains, which was clearly visible in the SEM images, is however not unique. Several thinner lamellae, which cannot be detected at the SEM scale, with the same orientation as the large lamella are observed within the olivine crystals (Figure 3c).

#### 2.2.3 Chemical Analyses by EDS

The chemical composition of the lamella has been determined with an EDS by scanning transmission electron microscopy spectrum image mode in the Thermo Fischer® Titan Themis TEM, operated at 300 kV. Quantification has been carried out on the basis of the stoichiometric oxides of the Van Cappellen and Doukhan (1994) method. The k‐factors (Cliff & Lorimer, 1975) have been obtained using stoichiometric oxide standard specimens which contain the studied phase elements.

**Figure 4:**
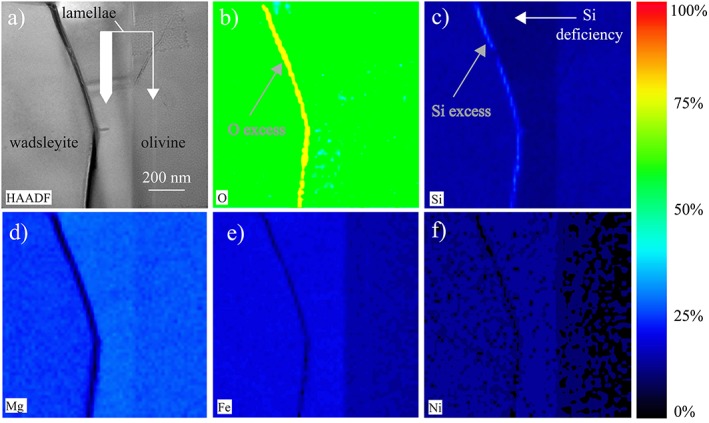
Energy‐dispersive X‐ray spectroscopy maps of wadsleyite, olivine and the lamellae, located in the same region: (a) High angular annular dark field image of a zone which contains a grain of wadsleyite (on the left), olivine (on the right), and the lamellae (in the middle). Energy‐dispersive X‐ray spectroscopy scanning transmission electron microscopy spectrum image of (b) oxygen, (c) silicon, (d) magnesium, (e) iron, and (f) nickel. The lamella is deficient in silicon (white arrow in c) relatively to both olivine and wadsleyite and enriched in iron and nickel relatively to olivine. It is rimmed by a silicon‐ and oxygen‐rich thin domain (indicated with a grey arrow in b and c). We can notice another thin lamella inside the olivine grain (thin white arrow in a), which width is below the resolution of the EDS analyzes.

Chemical maps are shown on Figure 4. Iron and nickel concentrations are higher in the lamella than in olivine, but, more importantly, the lamella shows significant deficiency in silicon compared to olivine. EDS analyses were performed inside and on both sides of the lamella. To ensure the accuracy of the quantifications, we have verified that the phase composition around the lamella matches with stoichiometric olivine and wadsleyite. We have found on both sides of the lamella, compositions of Mg_1.75_Fe_0.2_Ni_0.05_Si_0.99_O_4.01_ and Mg_1.89_Fe_0.1_Ni_0.01_SiO_4_ for wadsleyite and olivine, respectively. Both minerals display therefore a general composition of (Mg, Fe, Ni)_2_SiO_4_, with a (Mg+Fe)/Si ratio of 2. The composition of the lamella is Mg_1.99_Fe_0.21_Ni_0.05_Si_0.84_O_4_, which can also be written as (Mg, Fe, Ni)_13.8_Si_5.04_O_24_. It has therefore a (Mg+Fe)/Si ratio of 2.738. Next to the lamella, an ultrafine layer a few nanometers thick (grey arrow in Figures 4b and 4c) is enriched in silicon and oxygen. Quantification of this ultrafine layer is consistent with a SiO_2_ composition.

#### 2.2.4 Precession Electron Tomography in the TEM

Precession electron diffraction tomography was carried out on a FEI® Tecnai G2 20 operated at 200 kV and equipped with a NanoMEGAS Digistar precession device and an ORIUS 832 Gatan charge‐coupled device camera with 14‐bit dynamic range. The diffraction patterns were obtained using a defocused parallel beam and a selected area aperture of about 250 nm in diameter, smaller than the width of the lamellae. The lamella has been orientated in the microscope in such a way that the goniometer tilt axis is kept perpendicular to the interface plane during the tilt experiments only for the lamella that contributed to the collected diffraction patterns series. The electron diffraction pattern tilt series were collected by varying the tilt angle from ‐45° to +45° with an acquisition step of 1° (91 patterns for one tilt series). The precession angle was set to 1.2°, slightly higher than the tilt step in order to ensure a full coverage of the reciprocal space within the tilt range. During the tilt series acquisition, the position of the aperture with respect to the lamellae was checked every few degrees.

#### 2.2.5 Crystal Structure Determination and Refinements

Precession electron tomography data processing was performed using the PETS program (Palatinus, 2011). After peak hunting on all patterns, the value of the azimuthal angle (i.e., the angle between the horizontal axis and the projection of the tilt axis) was refined. After clustering, a difference vector space analysis was computed to produce a complete representation of the reciprocal space. The program JANA2006 was then used to find the unit cell (*a* = 5.9181(13) Å, *b* = 10.1141(9) Å, *c* = 14.3428(13) Å, and *V* = 858.5(2) Å^3^) and to refine the orientation matrix, allowing integration of the intensities for each pattern by PETS. At this step, Inorganic Crystal Structure Database as well as American Mineralogist Structure Database were consulted with a 5% tolerance on cell parameters. Since no results were returned, an ab initio structure solution was started. In a first step, a kinematical approach was used and intensities belonging to the same reflection on adjacent patterns were integrated together, leading to a list of intensities containing one value per hkl index with the corresponding estimated standard deviation. The reflection conditions are h0l, l=2n and hk0, *k*=2n, indicating the possible space group *Pmcb* (number 55, nonstandard setting of *Pbam*).

**Figure 5:**
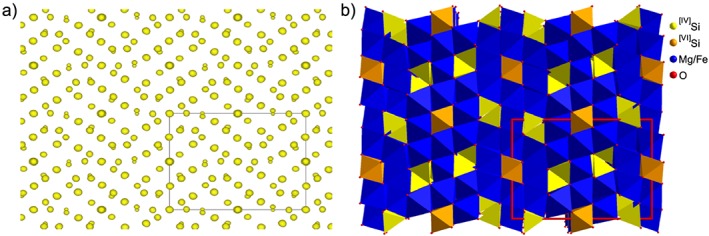
(a) [100] projection of the electron density isosurface of the structure solution. Note that the size of the “balls” is related to the number of electrons on the site, enabling both the identification of the atomic species and the site occupation refinement. (b) [100] projection of the proposed model in term of polyhedral pattern.

The structure was then solved ab initio using the charge flipping algorithm, as implemented in the program Superflip (Palatinus & Chapuis, 2007) with the chemical information provided by the EDS analysis (Mg_1.99_Fe_0.21_Si_0.84_O_4_). After several cycles of calculations, SUPERFLIP found a solution with an overall agreement factor (wR) around 20%, which might be high for X‐ray data, but is a rather good value for electron diffraction data considering the remaining dynamical diffraction effects. The [100] projection of the electron density isosurface of the structure solution is presented in Figure 5a, and its interpretation in term of atomic positions (taking into account common tetrahedral or octahedral geometries) is presented in Figure 5b. At this time, considering that (i) tetrahedral sites are occupied by silicon (with Si‐O distances between 1.62 and 1.70 A), (ii) “big” octahedral sites (distances between 1.98 and 2.23 A) are occupied by magnesium, and (iii) “small” octahedral site (6 distances around 1.81 A) is occupied by iron, an estimated chemical formula would be Mg_2.333_Fe_0.167_Si_0.667_O_4_.

However, it is now well established that the kinematical approach is not appropriate to determine the correctness of such a build structure (Palatinus, Petříček, & Correa, 2015; Roussel et al., 2014) and that dynamical effects are still present in the data even with beam‐precession. The previous model was therefore used as the starting one for dynamical refinement. This still emerging approach has already been used successfully a few times and is now recognized as a good alternative for accurate structure determination and refinement of submicronic samples (Colmont et al., 2016; Palatinus et al., 2017; Rondeau et al., 2019). After several cycles of refinements of both atomic parameters (positions, and thermal ones) and orientation‐thickness of the sample, encouraging agreement factors were obtained, supporting the opportunity to go further with this dataset. At this point, several possibilities were tested, such as the possibility to have mixed occupancy of Mg and Fe on big sites and only iron on small ones. This hypothesis leads to better agreement factors and to a chemical formula closest to the EDS‐determined one. However, the atomic displacement parameter of the small site (fully occupied by iron in this case) is abnormally high, indicating that this site “possesses too many electrons”. A way to decrease the number of electrons assigned on this site is to replace, partly or entirely, the iron (*Z*=26) by silicon (*Z*=14). The two hypotheses were tested, and both lead to close agreement factors (wR around 0.075) and this time reasonable thermal parameters. The refined chemical formulae are Mg_1.943_Fe_0.414_Si_0.809_O_4_ for a site occupied by 85% of Si and 15% of Fe and Mg_1.958_Fe_0.375_Si_0.833_O_4_ for a site occupied only by silicon, both very close the EDS one (Mg_1.99_Fe_0.21_Si_0.84_O_4_). In summary, whatever the chosen model, it is clear that this octahedral site is mostly occupied by silicon.

The dynamical refinement of the precession electron diffraction tomography data yields the structure presented in Figure 5, corresponding in fact to the already known anhydrous phase B (Anh‐B; Finger et al., 1989) but described with the alternative setting: *a*=0.59181(13) nm, *b*=1.43428(13) nm, and *c*=1.01141(9) nm). A matrix transformation allows to be in the same setting as Finger et al. (1989), which, for consistency with previously published studies, is used throughout the present paper. The final crystallographic data and refinement details are provided in Table S1 in the supporting information. Fractional coordinates and equivalent isotropic displacement parameters are given in Table S2, while selected interatomic distances are given in Table S3.

#### 2.2.6 EELS Analyses

In silicates, silicon is usually in tetrahedral sites (as in quartz, olivine, or wadsleyite, for instance), but with increasing pressure it can occupy octahedral sites (as in high‐pressure phases like stishovite or bridgmanite). To confirm the silicon coordination predicted by the structure refinement, we have characterized its oxygen environments. We have performed EELS analyses and more specifically energy loss near edge structure (ELNES) characterizations. Three ELNES spectra can be used to distinguish between tetrahedral and octahedral sites for silicon: the Si K‐edge, Si L2.3‐edge, and O K‐edge. We have focused our attention on the O K‐edge ELNES spectra. Actually, this edge has low core loss energy (near 530 eV) and offers noticeable changes in ELNES signatures from SiO_4_ to SiO_6_ (Sharp et al., 1996).

EELS analyses have been performed with a Thermo Fischer® Titan Themis TEM, operating at 80 kV to reduce electron beam damage. ELNES structures are analyzed with a monochromated STEM spectrum image mode, with an energy resolution of approximately 0.3 eV. In order to detect the ELNES details, the spectrometer energy dispersion was chosen to be 0.025 eV/channel.

**Figure 6:**
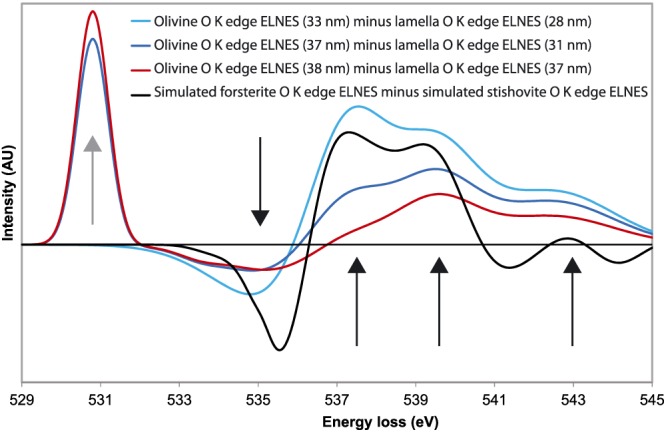
Experimental and simulated differential O K‐edge energy loss near edge structure (ELNES) spectra: Specimen degradation noted by a peak appearance of O_2_ cluster development (grey arrow at 530.8 eV). The signature of the differential spectra is composed of four peaks (black arrows near 535, 537.5, 539.5, and 543 eV) whose intensities depend on the thickness analyzed zones.

To highlight the differences between olivine ELNES structures (where silicon atoms are all in tetrahedral sites) and the ELNES structures of the phase in the lamella (where we suppose the occurrence of silicon atoms in octahedral sites), we have analyzed the “olivine//lamella” differential EELS spectra. The estimated thicknesses indicated in parenthesis on Figure 6 have been obtained by comparing energy‐filtered intensities to keep only the elastic electrons, with raw intensities, thanks to the following equation extracted from the Poisson law:

(1) $$I_{0}/I=\exp\left(-t/\lambda \right)$$

where *I*_0_ is the signal intensity obtained with elastic electrons, *I* is the raw signal intensity, *λ* is the mean free path, and *t* is the specimen thickness. From Tanuma et al. (1993), a mean free path of 84 nm has been estimated for olivine with a voltage of 80 kV.

It is worth mentioning a peak appearance at 530.8 eV, already noticed by Mkhoyan et al. (2006) and Garvie (2010) at approximately 531 and 530 eV, respectively. This peak growth has been interpreted as a structure damage leading to O_2_ cluster developments. The intensity normalization of differential ELNES spectra is done on the highest peak after the peak observed at 530.8 eV, that is, for an energy starting from 532 eV. Figure 6 shows the normalized differential O K‐edge ELNES spectra of olivine//lamella for different zones characterized by different thicknesses since this parameter has a strong influence on the spectra. Whatever the thickness, the lamella is less affected by O_2_ cluster developments than olivine, suggesting that the Si─O bonds within the lamella are stronger than in olivine. It can be noticed that there is no peak at 530.8 eV for the smallest thicknesses (≈30 nm), suggesting the impossibility to store O_2_ clusters in such thin zones. To compare these experimental results with literature, the simulated O K‐edge ELNES spectrum of forsterite (the pure magnesia pole of olivine) has been subtracted to the simulated O K‐edge ELNES spectrum of stishovite (see Figure 2 in Winkler et al., 2013 and Figure 4 in Kaneko et al., 1998, respectively) to obtain a simulated differential O K‐edge ELNES spectrum of « SiO_4_ minus SiO_6_ » with the same normalization process. The evolution of experimental differential spectra with the thickness is significant. From Figure 6, four peaks can be noticed, located near the same energies than the simulated differential spectrum ones, that is, 535, 537.5, 539.5, and 543 eV, respectively. The peak situated at 535 eV is negative, and the last peak (at 543 eV) is positive with a small intensity. The two other peaks are positive with medium intensities. Regardless of the studied zones thickness, approximately the same differential ELNES signatures are noted as the simulated ones. This confirms the hypothesis of the occurrence of silicon in octahedral sites in the lamella and hence the identification of Anh‐B.

## 3 Atomic Scale Modeling

**Figure 7:**
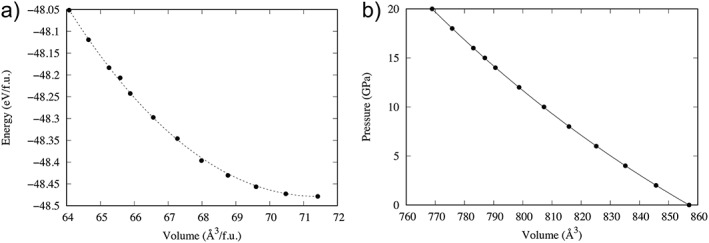
Atomic scale modeling of anhydrous phase B. (a) Energy as a function of the volume. (b) Equation of state, fit function according to the third‐order Birch‐Murnaghan equation.

To provide further information on the Anh‐B structure, calculations based on the density functional theory were performed using the VASP simulation package (Kresse & Hafner, 1993). All results are calculated using generalized gradient approximation (Perdew & Wang, 1992) with pseudopotential (of PAW type) based upon projector‐augmented waves (Blöchl, 1994, Kresse & Joubert, 1999). Outmost core radius for Mg, Si, and O atoms are 2, 1.9, and 1.52 au, respectively. Throughout this study, a single cutoff value of 600 eV was used for the plane wave expansion. The first Brillouin zone was sampled using a Monkhorst‐Pack grid (Monkhorst & Pack, 1976). For the Anh‐B orthorhombic unit cell, using a 6***×***4***×***4 mesh leads to convergence of the energy to better than 0.1 meV/atom. To compute the equation of state (EOS; Figure 7) and determine the elastic constants at a given pressure, we first calculate the equilibrium structure of a unit cell by minimizing the Hellman‐Feynman forces and stresses. Then, the elastic constants are computed by applying strains (magnitude ±1%) and calculating the resulting stress tensor.

At ambient pressure, the lattice parameters for the pure Mg end‐member of the Anh‐B (Mg_14_Si_5_O_24_ with 86 atoms in the unit cell) are 0.591, 1.429, and 1.014 nm. At 15 GPa, the lattice parameters are 0.574, 1.394, and 0.983 nm. The pressure‐volume calculations were fitted to a third‐order Birch‐Murnaghan EOS to yield *B*_0_=146.86 GPa and *B*′_0_=4.17. The bulk modulus *B*_0_ is in agreement with the experimental one from Crichton et al. (1999): *B*_0, exp_=151.5 GPa. We note however that its pressure derivative derived from experiments is slightly higher (*B*′_0, exp_=5.5). Nevertheless, our calculations are somewhat consistent with those reported by Ottonello et al. (2010) using a different density functional method. According to the EOS, the bulk modulus at 15 GPa is thus 209.45 GPa.

According to the computation of the elastic constant tensor at 15 GPa (Table 1), the bulk modulus defined as an average between Voigt and Reuss calculations is 209.21 GPa, self‐consistent with the value deduced from the EOS. The shear modulus is 119.7 GPa.

**Table 1:** Elastic Constants (GPa) and Average Moduli (GPa) of Anh‐B Computed at 15 GPa

C_11_	C_22_	C_33_	C_12_	C_13_	C_23_	C_44_	C_55_	C_66_
370.7	402.1	349.6	124.5	129.6	127.3	114.5	122.5	115.7
B_V_	209.4		B_R_	208.9				
G_V_	119.9		G_R_	119.5				

*Note. Pmcb* space group.

## 4 Seismic Properties

**Figure 8:**
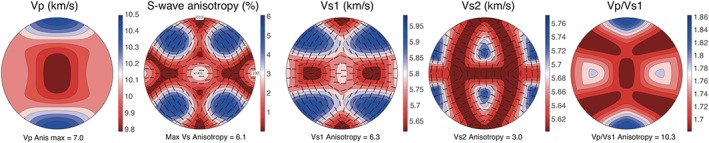
Seismic properties of anhydrous phase B at 0 K and 15 GPa calculated based on the lattice parameters and elastic constants calculated in this study using the MTEX open‐source Matlab toolbox (Mainprice et al., 2011). From left to right: *P* wave propagation anisotropy, *S* wave polarization anisotropy, fast *S* wave (S1) propagation anisotropy, slow *S* wave (S2) propagation anisotropy, and Vp/Vs1 ratio. Lower hemisphere stereographic projections in the crystal reference frame (indicated in the *S* wave polarization anisotropy plot).

The elastic constants calculated above can be used to assess the seismic properties of Anh‐B. At 0 K and 15 GPa, Anh‐B displays moderate seismic anisotropy (Figure 8), which is lower than the intrinsic anisotropy of olivine and wadsleyite (Mainprice, 2007). However, the geometry of anisotropy is similar to that of wadsleyite (Mainprice, 2007). The maximum *P* wave anisotropy is 7%, with the fastest *P* waves propagating parallel to [010] and the slowest, parallel to [001].

*S* wave polarization anisotropy has a complex pattern. The maximum *S* wave polarization anisotropy (6.1%) is observed for *S* waves propagating obliquely to all three main crystallographic axes, close to [111] directions. These *S* waves are polarized parallel to <uv0> directions. Low *S* wave polarization anisotropy (<2%) is observed for propagation directions along (100) and (010) planes, except parallel to [001] and [100], where intermediate polarization anisotropy values (2.5‐3%) and fast polarizations parallel to [100] are observed. Apparent *S* wave polarization isotropy is observed for propagation parallel to [010] or within approximately 25° of [100].

Vs1 velocity patterns show maximum velocities for propagation directions at low angle to <210> directions and minimum velocities for propagation directions parallel to [010] and <201>. When propagating within the (010) plane, Vs1 velocities are rather low and display a 90° periodicity. Low Vs1 propagation anisotropy (<3.5%) is observed within this plane, as well as within the (100) plane. The highest Vs1 propagation anisotropy is observed for propagation within the (001) plane. Vs2 velocities are low for propagation directions parallel to all three main crystallographic axes and minimum for propagation parallel to [010] and [001], as well as at approximately 20‐30° to the (100) plane and in the (010) plane at >30° to [100]. High Vs2 are observed for propagation in the (001) plane at approximately 30° to [100] and in the (100) plane at approximately 30° to [001].

Vp/Vs1 ratios are highly anisotropic. The highest ratios (1.86) are observed for propagation directions parallel to [010]. All propagation directions at >20° to [010] sample Vp/Vs1 ratios <1.8.

## 5 Discussion

This study describes a microstructural characterization with the TEM of a sample of San Carlos olivine partially transformed to wadsleyite at high‐pressure (14 GPa) and temperature (1400 °C). At the SEM scale, the transformation into wadsleyite (in the form of irregular domains at grain boundaries or inside grains) was readily observed. However, some lamellae with morphologies clearly distinct from the wadsleyite domains were also present. At the TEM scale, we have used the recently developed (Rauch et al., 2008, 2010) ACOM‐TEM technique to identify the phases on FIB sections containing all phases of interest. In this technique, a collection of electron diffraction spot patterns is acquired, while an area of interest of the sample is scanned by the electron beam. The recorded electron diffraction spot patterns are compared with templates calculated for all possible orientations of the phases expected to be present in the area of interest. We have calculated templates corresponding to olivine, wadsleyite, and ringwoodite using crystallographic data from Birle et al. (1968), Horiuchi and Sawamoto (1981), and Hazen et al. (1993), respectively. The olivine matrix and wadsleyite grains resulting from the HP‐HT treatment were readily indexed. The lamella identified at the SEM scale was not recognized to be one of those three phases.

### 5.1 Identification of Anh‐B

**Figure 9:**
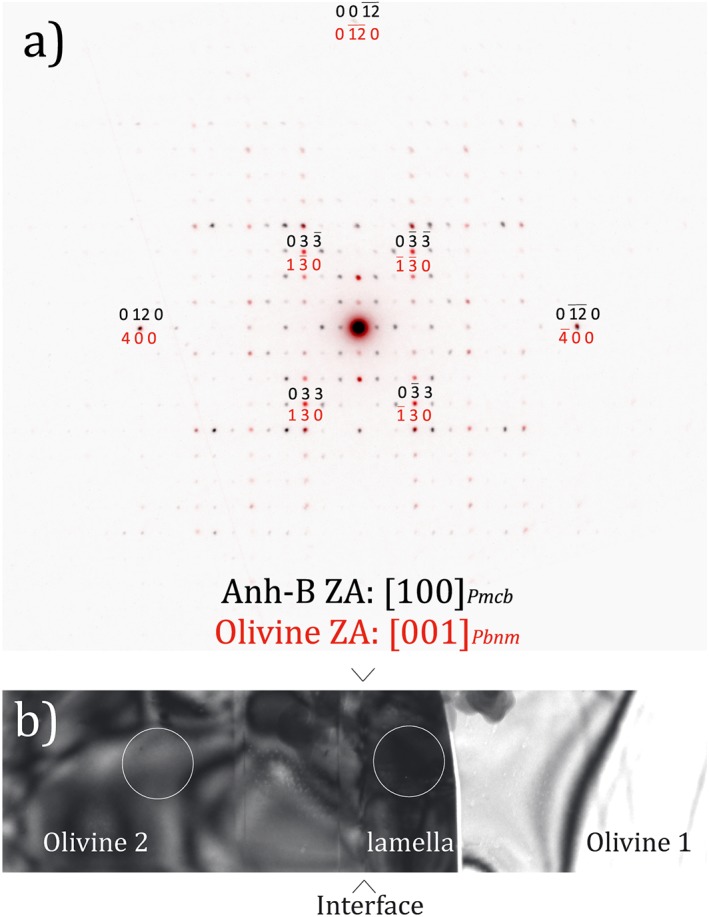
(a) Superimposition of the diffraction patterns acquired on the anhydrous phase B (Anh‐B) lamella (colored in black) and in olivine 2, near the lamella (colored in red). The exact locations are shown in (b). A precession angle of 3° has been applied to the electron beam to get quasi‐kinematic conditions. A superstructure can be noted between the [100]_ol_ direction of olivine and the [010]_Anh‐B_ direction of Anh‐B (3 × 0.48 nm ≈ 1.43 nm); and a correspondence is observed between the [010]_ol_ direction of olivine and the [001]_Anh‐B_ direction of Anh‐B. (b) Transmission electron microscopy bright‐field corresponding to the acquisition of the diffraction patterns of (a). The interface between the lamella and olivine 2 is edge‐on and located between the arrows. It corresponds to (100)_ol_ plane in olivine and (010)_Anh__‐__B_ in Anh‐B. The circles (250 nm in diameter) represent the position of the selected‐area aperture for acquisition of the diffraction patterns of (a).

The observation of the FIB section using diffraction contrast (bright‐ and dark‐fields) yielded additional information. First, the grains indexed as wadsleyite by the ACOM‐TEM indeed contain the characteristic stacking faults (Figure 3a) usually found in this mineral. The 320‐nm‐thick lamella (observed at the SEM) shows no defects. At the TEM, we see that this lamella located at the interface between two olivine grains is not unique in the FIB section. Some much thinner (10‐20 nm) lamellae with the same orientation are found in the olivine grain at the bottom of Figure 3. We assume that all those lamellae are constituted of the same phase. This assumption has not been demonstrated but is at least compatible with the chemical analyses performed (see Figure 4). A striking observation is the existence of topotactic relationships between the lamellae and the parent olivine. This is illustrated by Figure 3b, which shows that both the lamella and olivine can be imaged in weak‐beam dark‐field with very close diffraction vectors (*g* = 004 for olivine and *g* = 400 for Anh‐B). On this figure, one can see the network of interfacial dislocations, which accommodate the small lattice parameter difference between the two structures in contact. The topotactic relationship between the two structures is along the (100)_ol_ plane of olivine, which corresponds to the (010)_Anh‐B_ plane of Anh‐B. Indeed, the crystal structures of olivine and Anh‐B are very close. The [100]_Anh‐B_ direction of Anh‐B in *Pmcb* corresponds to [001]_ol_ in olivine. The [010]_Anh‐B_ direction of Anh‐B corresponds to [100]_ol_ in olivine and represents a superstructure of it. The [001]_Anh‐B_ direction of Anh‐B corresponds to [010]_ol_ in olivine. These relationships are well illustrated when superimposing diffraction patterns from both phases as shown on Figure 9. Additional examples (from calculated diffraction patterns) are presented in Figure S4.

The determination of crystal structure models is now possible from electron diffraction data (Kolb et al., 2007, 2008; Palatinus, Corrêa, et al., 2015; Palatinus, Petříček, & Correa, 2015; Wan et al., 2013). This allows, as in the present case, to characterize phases with volumes so small that single‐crystal X‐ray diffraction is precluded. Here some lamellae are large enough for selected area electron diffraction to be performed without sampling the matrix. The analyzed volume is smaller than 0.005 μm^3^. To get the maximum coverage of the reciprocal space, which is necessary to produce reliable structure model, we use here precession electron diffraction tomography, which combines precession electron diffraction (Vincent & Midgley, 1994) and electron diffraction tomography. For the tomography, we use a tilt range of **±** 45° in the microscope. Combined with the use of precession, this allows a broad coverage of the reciprocal space, which is satisfactory for three‐dimensional structure determination. In precession electron diffraction, the electron beam is tilted away from the optical axis of the microscope and precessed on a conical surface with a semi‐angle usually between 1 and 3° (here 1.20°). This technique has two advantages: precession electron diffraction patterns are less dynamical and they display a larger number of reflections. Despite that, taking into account dynamical effects is important for the accurate structure refinement (Palatinus, Petříček, & Correa, 2015). The crystal structure model obtained from this data processing is characterized by having part of silicon in sixfold coordination.

Since the discovery of stishovite (Chao et al., 1962; Stishov & Popova, 1961), it is well known that with increasing pressure (between 8 and approximately 30 GPa) most crustal silicates undergo phase transformations to new structures with sixfold coordinated silicon (Finger & Hazen, 1991). In the Earth, this transformation is achieved below 670‐km depth. This is however usually not observed in Mg_2_SiO_4_ since all high‐pressure polymorphs known of olivine involve fourfold coordinated silicon. Since this characteristic (silicon in sixfold coordination in the crystal structure model) was critical in the identification of the phase, we decided to carry on an ELNES characterization at the O K‐edge, which did confirm the presence of sixfold coordinated silicon. The structure model obtained from the structure refinement, which corresponds to Anh‐B, is thus in agreement with all our observations. It also fully explains the chemical composition measured since, starting from an olivine composition, the formation of Anh‐B leads to an excess of silica (equation (2)), which was indeed visible on the chemical maps (Figures 4b and 4c). At the pressure considered here, the thin silica layer observed on one side of the lamella is likely stishovite, although we have not checked the crystal structure.

### 5.2 Stability and Conditions of Formation

The formation of Anh‐B was not expected and is a priori surprising since the annealing was carried out in the stability field of wadsleyite (which is indeed observed). To synthesize Anh‐B, Ganguly and Frost (2006) started with oxide mixtures, which corresponded to a composition enriched in MgO compared to forsterite. In this system, our conditions (14 GPa, 1400 °C) would indeed favor the formation of Anh‐B (see Figure 1 of Ganguly & Frost, 2006, and also the results of calculations from Ottonello et al., 2010, from their Figure 6). This can further be verified by computing the enthalpy change corresponding to the destabilization of Mg_2_SiO_4_ units into Anh‐B and stishovite (St) following the reaction:

(2) $$7.{\mathrm{Mg}}_{2}\mathrm{Si}O_{4}\left(\mathrm{Fo},\mathrm{Wa},\mathrm{Ri}\right)\to {\mathrm{Mg}}_{14}{\mathrm{Si}}_{5}O_{24}\left(\mathrm{Anh}-B\right)+2.\mathrm{Si}O_{2}\;\left(\mathrm{St}\right)$$

According to the energy computed here for Anh‐B (Figure 7) and combining with those computed by Hernández et al. (2015) using the same set of pseudo potentials, we verified that above 14 GPa, the mixture Anh‐B + stishovite is indeed a little bit more stable than forsterite but less stable than wadsleyite. Ganguly and Frost (2006) have also calculated the possible reactions (Fo/Wads/Ring) = Anh‐B + Stishovite. They show that at 1400 °C, the formation of Anh‐B is indeed not expected. To grow Anh‐B, Kojitani et al. (2017) and Yuan et al. (2018) started from mixtures having the stoichiometric composition of Anh‐B. Alternatively, Anh‐B has first been reported by Herzberg and Gasparik (1989) from melting experiments at 16.5 GPa on chondritic material, and in the run product of melting experiments of forsterite at 16.5 GPa, 2380°C (Presnall & Gasparik, 1990). To the best of our knowledge, the only report for direct formation of Anh‐B from stoichiometric olivine composition has been done by Hazen et al. (1992) in a Fe‐bearing system (Fo80). The unexpected stability of Anh‐B was attributed to the presence of Fe.

Most of the thermodynamic approaches (e.g., Ottonello et al., 2010, or the discussion above regarding the reaction enthalpy of equation (2)) do not describe however an important stage, which is nucleation. During solid‐state transformation when a product phase nucleates within the parent phase, the energy balance does correspond not only to the volume free energy but also to the interfacial energy. A combination of the two energies constrains the nucleation barrier. In the present case, we see that unlike wadsleyite, which forms irregular domains (inside olivine grains or at grain boundaries) with no preferential orientations with olivine, Anh‐B nucleates as thin lamellae. Most of them are inside the grains. We note that the larger one occurs at a grain boundary, which exhibits a specific orientation. This orientation yields a very small interfacial energy, which corresponds to the dislocation network observed on Figure 3b. For that reason, the energy barrier for nucleation of Anh‐B is likely smaller than for wadsleyite. As the phase grows, the contribution of interfacial energy becomes smaller and growth of the thermodynamically stable wadsleyite dominates. Another factor also probably inhibits the growth of Anh‐B. It must be remembered that only one side of the lamella can benefit from this low‐energy interface. On the other side grows the stishovite layer. We have no hint as to whether the Anh‐B/stishovite and stishovite/olivine interfaces have high or low interfacial energies. Also, besides equilibrium thermodynamics considerations, this decomposition into Anh‐B and stishovite must slow down as growth proceed since it requires diffusion of O and most importantly of Si across the Anh‐B lamella toward the stishovite lamella.

To summarize, we have enough elements to rationalize the observation of Anh‐B in our experiments and understand that during a short interval, which corresponds to the nucleation stage, nucleation of Anh‐B can compete with the one of wadsleyite. However, one can expect, in agreement with the observed microstructure (see Figure 1), that in the transition zone, growth of wadsleyite rapidly exceeds growth of Anh‐B. The situation is different if some MgO‐enriched or SiO_2_‐depleted regions exist. Several studies have suggested the existence of P‐T conditions where Anh‐B might be stable (Ganguly & Frost, 2006; Ottonello et al., 2010; Yuan et al., 2018). Our study shows that transformation from olivine can be facilitated due to low nucleation energy barriers. Also, one can expect that crystal preferred orientations of Anh‐B could be inherited from those of olivine (if they exist).

### 5.3 Elastic and Seismic Properties, Occurrence in the Mantle, and Detectability

Anh‐B might form in places characterized by an enrichment of MgO or a depletion of SiO_2_. Yuan et al. (2018) suggest that “MgO‐rich compounds might be produced through incongruent melting of both anhydrous and hydrous peridotites” or from the reduction of subducted carbonates. Ganguly and Frost (2006) have proposed that an assemblage Anh‐B + stishovite might develop in cold slabs. To evaluate whether the presence of Anh‐B can be detected through a seismic signature, we have calculated its elastic and seismic properties. The elastic properties of Anh‐B can be compared to those of forsterite calculated at the same pressure, 15 GPa (Durinck et al., 2005). With a bulk modulus 5% larger and a shear modulus 20% larger, Anh‐B appears slightly stiffer than forsterite. Anh‐B is also more isotropic from the elastic point of view. Taking the orientation relationships between Anh‐B and olivine into account, one can compare their elastic constants. In olivine, the three longitudinal moduli are significantly different: C_22_ is 37% softer than C_11_ and C_33_ is 27% softer than C_11_. In Anh‐B, C_33_ (equivalent to C_22_ in olivine) is only 13% softer than C_22_ (equivalent to C_11_ in olivine) and C_11_ (equivalent to C_33_ in olivine) is only 8% softer than C_22_. For both structures, the off‐diagonal moduli (C_12_, C_13_ and C_23_) exhibit very similar values. Concerning the shear moduli, in olivine, C_44_ is 10% softer than C_55_ and 15% softer than C_66_. In Anh‐B, the situation is comparable, but with an opposite variation since C_55_ (equivalent to C_44_ in olivine) is 7% stiffer than C_44_ and 6% stiffer than C_66_. This explains that Anh‐B displays only a moderate seismic anisotropy. Moreover, although it is less anisotropic, the seismic anisotropy pattern of the Anh‐B crystal is similar to the wadsleyite one (Mainprice, 2007). It seems thus difficult to observe a clear seismic signature of the presence of Anh‐B in MgO‐rich or SiO_2_‐depleted regions of the transition zone. If Anh‐B forms from olivine in cold slabs, as proposed by Ganguly and Frost (2006), the strongest signal in terms of seismic anisotropy may result from the presence of stishovite, which is one of the most anisotropic phases in the mantle (especially for *S* waves; see Mainprice et al., 2000). The volume fraction of stishovite is however expected to be very small and significant crystal preferred orientations would be necessary for it be detected. As discussed above, they could be inherited from those in olivine.

## 6 Conclusions

Here we demonstrate that structure refinement can be performed from processing three‐dimensional precession electron diffraction data acquired on sample volumes smaller than 0.005 μm^3^ of a high‐pressure phase. The technique produces a structure model, which is compatible with the chemical composition measured in the TEM by EDS and for which the presence of six‐coordinated silicon has been confirmed by EELS analyses.

In this study, we document the formation of Anh‐B (and stishovite) in San Carlos olivine at *P*=14 GPa and *T*=1400 °C. At these conditions, the stable phase would be wadsleyite, and indeed, we do see the formation of wadsleyite. In contrast to wadsleyite that occur both on grain‐boundaries and inside of grains with little topotactic relationships to the host (olivine), Anh‐B crystals show strong topotactic relationships to parent olivine grains. This suggests that in olivine, Anh‐B (and stishovite) can form due to kinetic reasons (lower nucleation energy barrier) as a meta‐stable phase.

## Supporting information

Supporting Information S1
