# Supplementary material for: Anhydrous Phase B: Transmission Electron Microscope Characterization and Elastic Properties
Source: Geochem Geophys Geosyst. 2019 Aug 14;20(8):4059–72. doi: 10.1029/2019GC008429 (PMC6853247; doi:10.1029/2019GC008429)
Supplement: Supplementary file 1 — Supporting Information S1 [file GGGE-20-4059-s001.pdf]

Geochemistry, Geophysics, Geosystems (G-Cubed)

Supporting Information for

Anhydrous Phase B: Transmission Electron Microscope Characterization and Elastic Properties

A. Addad[1], P. Carrez[1], P. Cordier[1], D. Jacob[1], S-i. Karato[2], A. Mohiuddin[2], A. Mussi[1], B.C. Nzogang[1], P. Roussel[3], A. Tommasi[4]

[1]Univ. Lille, CNRS, INRA, ENSCL, UMR 8207 - UMET - Unité Matériaux et Transformations, F-59000 Lille, France

[2]Yale University, Department of Geology and Geophysics, New Haven CT 06520-8109, USA.

[3]Univ. Lille, CNRS, Centrale Lille, ENSCL, Univ. Artois, UMR 8181 - UCCS - Unité de Catalyse et de Chimie du Solide, F-59000 Lille, France

[4]Univ. Montpellier, CNRS, Geosciences Montpellier, F-34095 Montpellier, France

Contents of this file

Figures S1 to S6
Tables S1 to S3

Introduction

- Figures S1 to S3 are complements of Figure 2 (phase map and inverse pole figures along two additional directions)
- Figure S4a corresponds to the calculated diffraction patterns corresponding to Figure 9. Figures S4b and c are additional calculated diffraction patterns illustrating crystallographic relationships between olivine and anhydrous phase B (Anh-B).
- Figures S5 and S6 compare the crystal structure of olivine and anhydrous phase B (Anh-B) along some low-index crystallographic directions.
- Tables S1 to S3 are final crystallographic data and details from the crystal structure determination and refinements performed on Anh-B in this study.

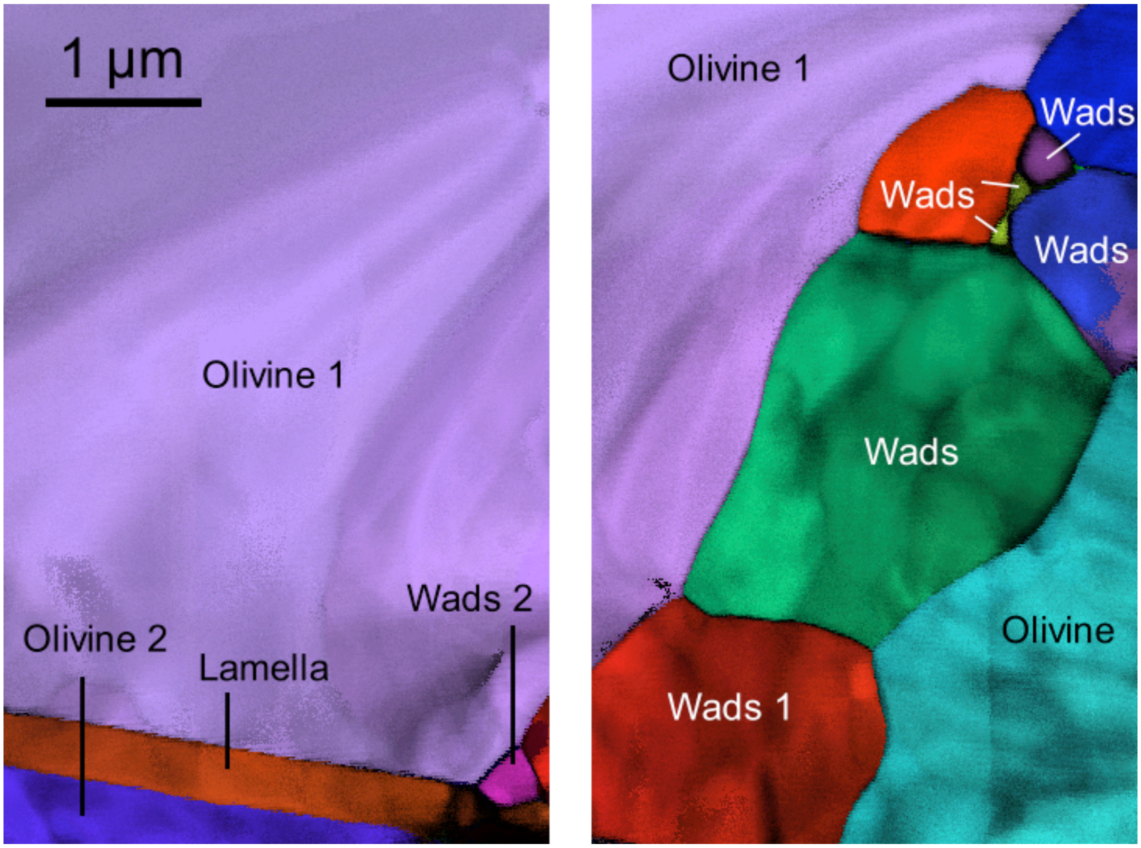

Figure S1. Supplementary figure to Figure 2. The inverse pole figures represented here correspond to the horizontal direction superimposed with the reliability maps. The color code is provided with Figure 2.

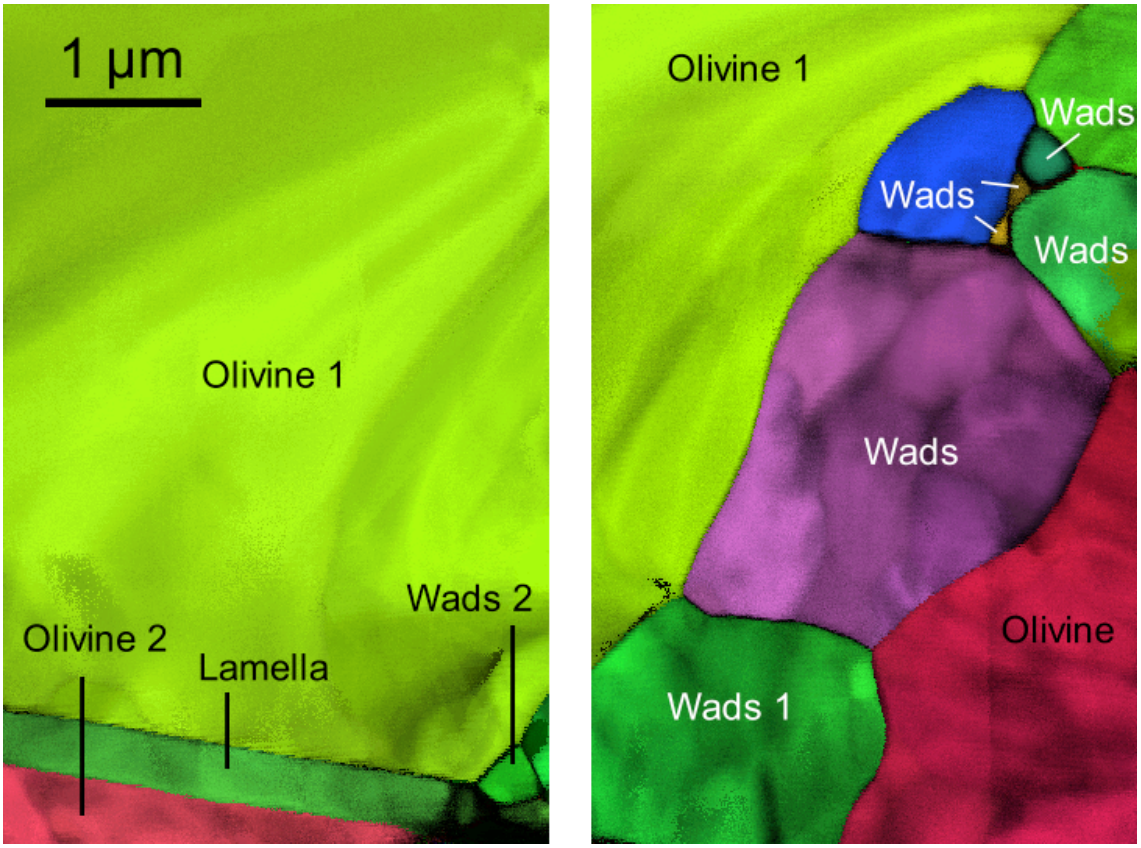

Figure S2. Supplementary figure to Figure 2. The inverse pole figures represented here correspond to the direction normal to the plane of representation, superimposed with the reliability maps. The color code is provided with Figure 2.

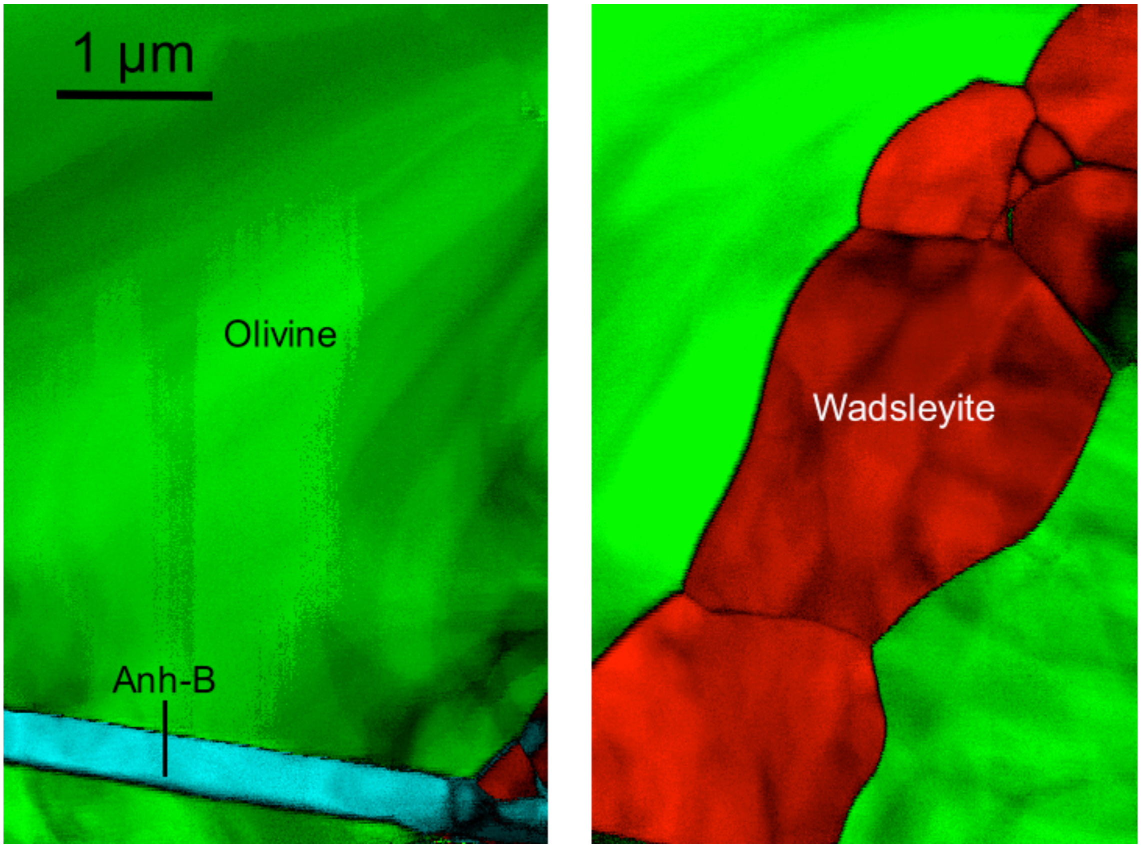

Figure S3. Supplementary figure to Figure 2. Phase maps, superimposed with the reliability maps. Color code: olivine is green; wadsleyite is red; Anh-B is blue.

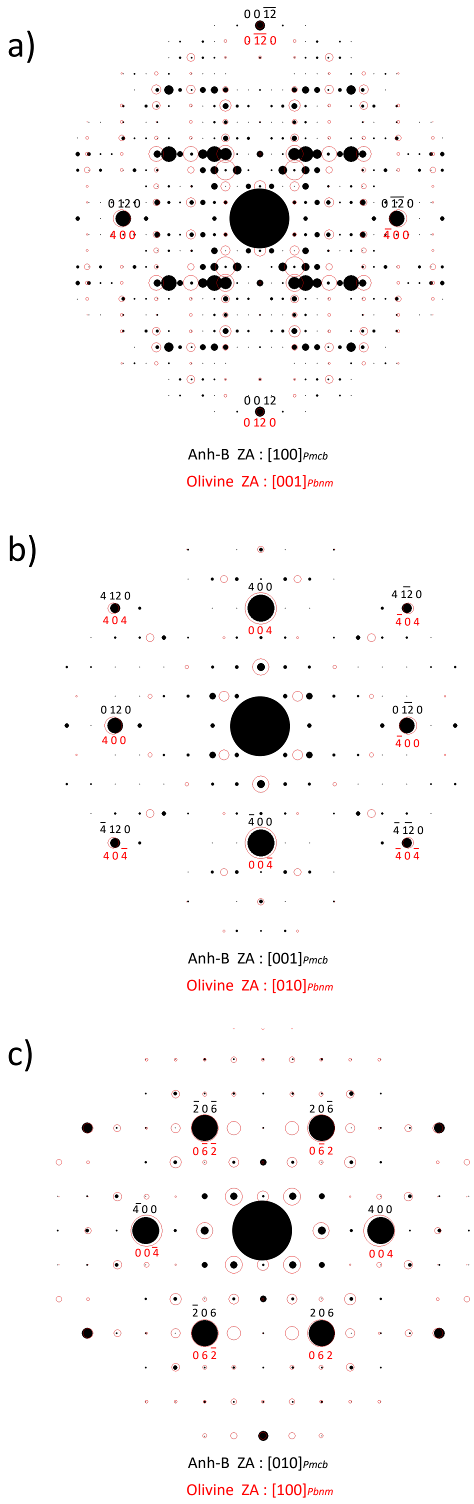

Figure S4. Superposition of calculated electron diffraction spot patterns of Anh-B and olivine along some low-index crystallographic directions.

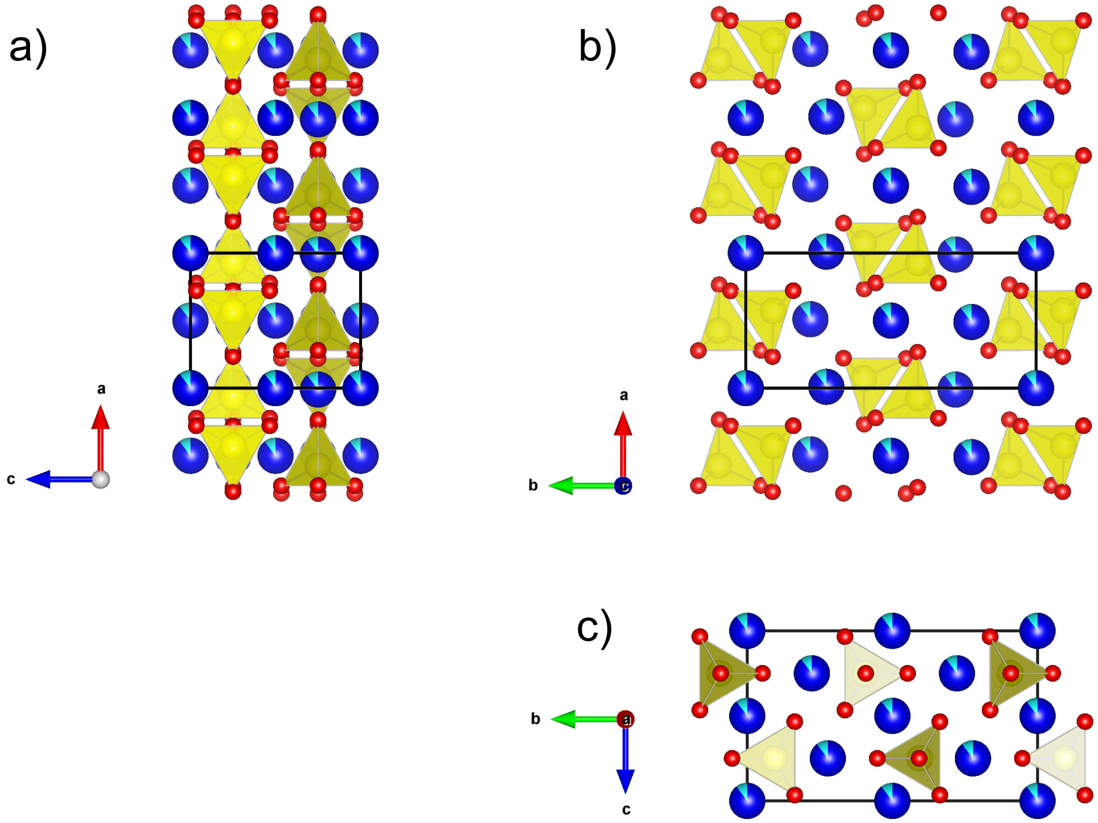

Figure S5. Crystal structure of olivine (Birle et al., 1968) represented along a) $[0\bar{1}0]$, b) $[001]$ and c) $[100]$. Polyhedra are represented for Si only (yellow). Oxygens are red, Magnesium, iron are blue.

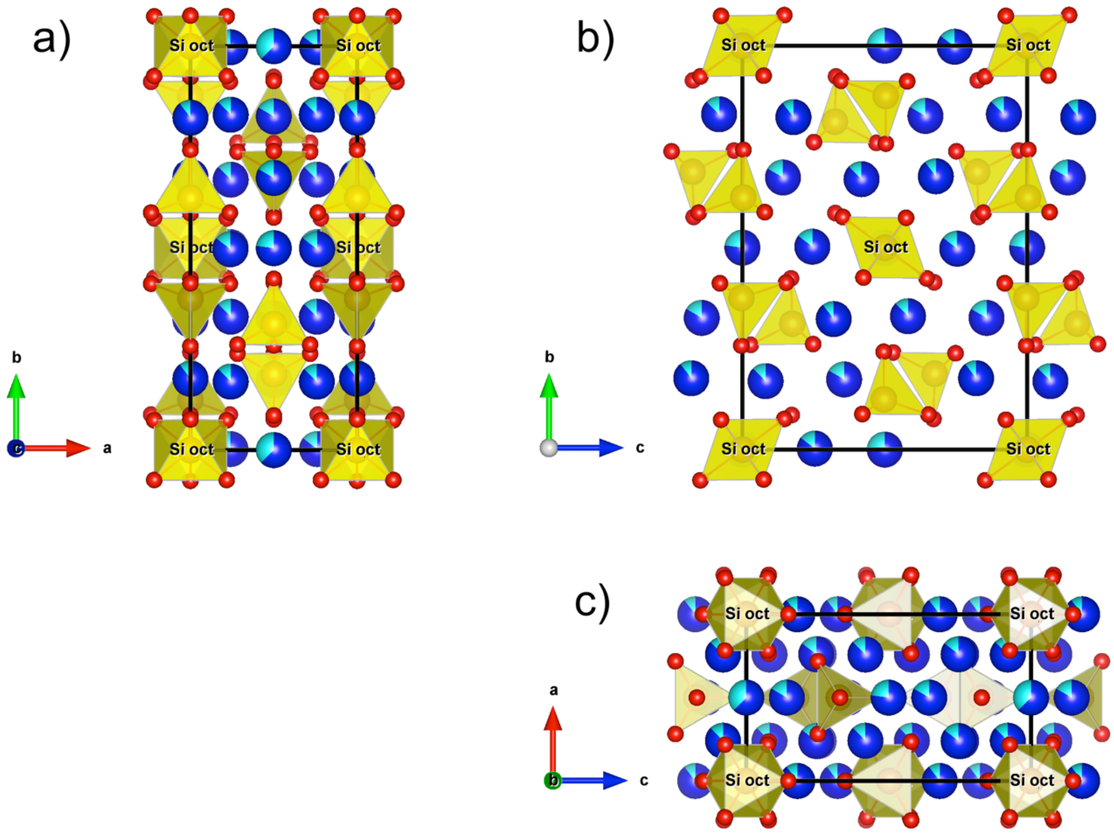

Figure S6. Crystal structure of Anh-B (our model) represented along a) $[001]$, b) $[\bar{1}00]$ and c) $[010]$. Polyhedra are represented for Si only (yellow). Oxygens are red, Magnesium, iron are blue.

Crystal data	
Crystal system	Orthorhombic
Space group	*Pmcb* (no. 55)
Unit-cell dimensions (Å)	a = 5.9181(13) Å
	b = 14.3428(13) Å
	c = 10.1141(9) Å
Volume ($Å^3$)	858.5(2) $Å^3$
Z	12
Chemical formula	$Mg_{1.958}Fe_{0.375}Si_{0.833}O_4$
D (calc.)	3.6195 $g.cm^{-1}$
Radiation, Å	0.0251
Temperature, K	293
Number of frames	91
Range of data collection, °	-45:+45
Tilt step, °	1
Precession angle, °	1.2
h,k,l range	-4:4, -18:18, -12:12
g max , S g max (matrix), S g max (refine), R Sg	2 , 0.01 , 0.1 , 0.4
Measured reflections	7013
Observed reflections (I ≥ 3σ(I))	3284
Thickness (nm, refined)	441(2)
R obs , wR obs	0.0707 , 0.0717
R all , wR all	0.1482 , 0.0752

Table S1. Crystal parameters, data collection and structure refinement parameters.

Type	occ	x	y	z	Uiso (A2)
Si1	1	0	0	0	0.0037(5)
Si2	1	0.5	0.31200(12)	0.17529(16)	0.0104(7)
Si3	1	0	0.37567(12)	0.99692(16)	0.0109(7)
Mg1<br>Fem1	0.6304(15)<br>0.370	0.5	0	0.5	0.0072(4)
Mg2<br>Fem2	0.831(7)<br>0.169	0.5	0.17379(12)	0.35737(16)	0.0161(7)
Mg3<br>Fem3	0.770(11)<br>0.230	0.5	0	0	0.0115(7)
Mg4<br>Fem4	0.854(6)<br>0.146	0.2398(3)	0.00253(8)	0.25436(11)	0.0123(5)
Mg5<br>Fem5	0.904(6)<br>0.096	0	0.17678(11)	0.82069(15)	0.0114(9)
Mg6<br>Fem6	0.866(6)<br>0.1344	0.2415(3)	0.16961(8)	0.08162(10)	0.0091(4)
O1	1	0	0.91331(16)	0.3467(2)	0.0119(6)
O2	1	0	0.57587(16)	0.3529(2)	0.0117(4)
O3	1	0	0.24229(15)	0.4962(2)	0.0112(4)
O4	1	0.5	0.08580(16)	0.1735(2)	0.0087(6)
O5	1	0.5	0.42397(14)	0.1710(2)	0.0115(6)
O6	1	0.5	0.75953(16)	0.4705(2)	0.0119(6)
O7	1	0.2359(4)	0.08804(12)	0.42119(14)	0.0133(4)
O8	1	0.2172(4)	0.42610(11)	0.42521(15)	0.0154(6)
O9	1	0.2820(4)	0.76140(12)	0.25137(16)	0.0112(6)

Table S2. Positional parameters.

Si1-O2^{i}	1.843(2)	(i)	-x,-y+1/2,z-1/2
Si1-O2^{ii}	1.843(2)	(ii)	-x,y-1/2,-z+1/2
Si1-O8^{i}	1.830(2)	(iii)	x,y-1/2,-z+1/2
Si1-O8^{ii}	1.830(2)	(iv)	x,-y+1/2,z-1/2
Si1-O8^{iii}	1.830(2)	(v)	-x+1,y-1/2,-z+1/2
Si1-O8^{iv}	1.830(2)	(vi)	-x,y-1/2,-z+3/2
		(vii)	-x,-y+1/2,z+1/2
Si2-O5	1.606(3)	(viii)	x,-y+1/2,z+1/2
Si2-O6^{v}	1.655(3)	(ix)	-x+1,-y+1/2,z+1/2
Si2-O9^{v}	1.656(2)	(x)	x,-y,-z+1
Si2-O9^{iii}	1.656(2)	(xi)	-x+1,-y,-z+1
		(xii)	-x+1,y,z
Si3-O1^{vi}	1.671(3)	(xiii)	x,-y+1,-z+1
Si3-O3^{vii}	1.692(3)	(xiv)	x,-y,-z
Si3-O7^{vii}	1.675(2)	(xv)	-x+1,-y+1/2,z-1/2
Si3-O7^{viii}	1.675(2)	(xvi)	x,y-1,z
		(xvii)	-x,-y+1,-z+1
Mg1-O5^{ix}	2.045(2)	Mg2-O4	2.247(3)
Mg1-O5^{v}	2.045(2)	Mg2-O6^{xiii}	1.986(3)
Mg1-O7	2.162(2)	Mg2-O7	2.091(2)
Mg1-O7^{x}	2.162(2)	Mg2-O7^{xii}	2.091(2)
Mg1-O7^{xi}	2.162(2)	Mg2-O9^{v}	2.110(2)
Mg1-O7^{xii}	2.162(2)	Mg2-O9^{iii}	2.110(2)
Mg3-O4	2.143(2)	Mg4-O1^{xvi}	2.127(2)
Mg3-O4^{xiv}	2.143(2)	Mg4-O2^{ii}	2.073(2)
Mg3-O8^{xv}	2.121(2)	Mg4-O4	2.113(2)
Mg3-O8^{v}	2.121(2)	Mg4-O5^{v}	2.052(2)
Mg3-O8^{iii}	2.121(2)	Mg4-O7	2.0860(19)
Mg3-O8^{iv}	2.121(2)	Mg4-O8^{iii}	2.1254(19)
Mg5-O1^{xiii}	2.130(3)	Mg6-O2^{ii}	2.071(2)
Mg5-O3^{vii}	2.121(3)	Mg6-O3^{i}	2.094(2)
Mg5-O8^{vii}	2.224(2)	Mg6-O4	2.156(2)
Mg5-O8^{viii}	2.224(2)	Mg6-O6^{v}	2.069(2)
Mg5-O9^{xiii}	2.026(2)	Mg6-O8^{iv}	2.0994(19)
Mg5-O9^{xvii}	2.026(2)	Mg6-O9^{iii}	2.155(2)

Table S3. Interatomic distances.
